# Validation of femoral pressure-derived assessment of effective arterial elastance in porcine ischemic cardiogenic shock

**DOI:** 10.3389/fcvm.2026.1815870

**Published:** 2026-07-21

**Authors:** Oskar Kjærgaard Hørsdal, Nigopan Gopalasingam, Kristoffer Berg-Hansen, Roni Nielsen

**Affiliations:** 1 Institute of Clinical Medicine, Aarhus University, Aarhus, Denmark; 2 Department of Cardiology, Aarhus University Hospital, Aarhus, Denmark

**Keywords:** acute coronary syndromes, afterload, arterial elastance, cardiogenic shock, hemodynamics, pressure–volume loops

## Abstract

**Background:**

During ischemic cardiogenic shock (CS), increased afterload overburdens the damaged myocardium and may worsen outcomes. The measure, effective arterial elastance (Ea), integrates systemic vascular resistance, arterial compliance, and heart rate, and reflects left ventricular (LV) afterload. While Ea is traditionally derived from invasive LV pressure–volume (PV) analysis, a simplified bedside formula (0.9 × systolic arterial pressure/stroke volume) has been validated in stable intensive care patients. Its accuracy in CS, characterized by rapidly evolving hemodynamics, remains unknown.

**Methods:**

In total, 20 female Danish Landrace pigs were instrumented with a femoral arterial catheter, pulmonary artery catheter (PAC), and LV PV catheter. CS was induced by microsphere embolization of the left main coronary artery. Ea was estimated from femoral arterial pressure and stroke volume measured with the PAC and compared with PV-derived Ea as a reference. Ea was measured at healthy baseline, CS onset, 60 minutes of untreated CS and after a dobutamine infusion. Agreement was evaluated with Pearson correlation and Bland–Altman analysis.

**Results:**

At baseline, onset of CS, and at established CS, correlations between the estimated and the PV-derived Ea remained strong (*r* = 0.68–0.74, all *P* ≤ 0.002). During dobutamine infusion, correlation persisted (*r* = 0.63, *P* = 0.020). Bland–Altman analysis showed negligible bias at healthy baseline and CS onset, slight underestimation during established CS, and modest overestimation during dobutamine-treated CS, with the widest limits of agreement observed during established untreated CS.

**Conclusions:**

Estimated Ea reliably reproduced PV-derived Ea during disease states. This simple bedside measure may enable continuous monitoring of afterload in ischemic CS without LV catheterization.

## Background

Cardiogenic shock (CS) following myocardial ischemia remains a syndrome with high in-hospital mortality. CS is commonly caused by coronary occlusion, resulting in abrupt loss of left ventricular (LV) contractile force and stroke volume (SV). To maintain end-organ perfusion, reflex sympathetic vasoconstriction occurs. This increases systemic vascular resistance (SVR) (1) but at the cost of increased LV afterload and diminished forward blood flow. These hemodynamic abnormalities are complex and need to be reliably characterized and monitored for appropriate hemodynamic supportive treatment. In CS, elevated afterload overburdens damaged myocardium and is associated with adverse outcomes, increased oxygen consumption, and aggravation of heart failure (2). Furthermore, vasoactive drugs used postcardiac arrest or during treatment of CS may alter LV afterload. Inopressors such as dopamine or norepinephrine may increase afterload, which may further aggravate cardiac function (3), while inodilators such as dobutamine or milrinone may decrease afterload. Thus, reliable estimation and monitoring of afterload hold potential to improve patient care.

For the assessment of afterload, the effective arterial elastance (Ea) is of particular interest (4, 5) as it incorporates SVR, arterial compliance, and heart rate (HR). Ea is traditionally measured using an invasive pressure–volume (PV) catheter in the LV and is considered a more precise reflection of LV afterload compared with arterial pressure or SVR (6, 7). However, LV catheterization is still highly specialized and not yet utilized in clinical practice, and thus, cannot be routinely used for assessment of Ea. Consequently, other methods for Ea estimation are needed. Ea can be approximated as the LV end-systolic pressure (ESP) to SV ratio, and thus, needs a reliable method of estimation of ESP. Therefore, translating the Ea concept to the bedside may suffer from approximations, including the estimation of ESP from arterial pressure. The literature lacks validation of such an estimation of Ea in patients with CS; however, recently, a bedside formula has been validated in hemodynamically stable intensive care patients using femoral arterial pressure (8). Yet the performance of this formula under the extreme, rapidly evolving hemodynamics of ischemic CS and vasoactive treatment is unknown. Hence, a knowledge gap exists regarding the value of bedside estimated Ea (Ea_estimated_) as a real-time surrogate of afterload during the precipitous decline from normal hemodynamics to overt CS when SV, SVR, and HR all change simultaneously.

Here, we present the first systematic validation of the bedside Ea estimation in a well-established large-animal CS model. We compare the estimated Ea calculated from femoral arterial pressure and SV measurement with the reference Ea derived from simultaneous high-fidelity LV PV measurements in animals in a healthy state and following ischemic CS.

## Methods

### Study design

In total, 20 female Danish Landrace pigs were examined. The study was conducted after obtaining the necessary authorization from the Danish National Animal Experiment Inspectorate. This study was conducted using animals that were part of a previously established research protocol in our laboratory at Aarhus University Hospital, ensuring maximum statistical power while adhering to the 3R principles aimed at minimizing animal use in research (9–11). The anesthesia protocol is described elsewhere (9–11). Ischemic CS was induced through repeated injections of polyvinyl microspheres (Contour, Boston Scientiﬁc, USA) into the left main coronary artery. CS was considered present with a 30% reduction in cardiac output (CO), measured using thermodilution, or with a 30% reduction in the perfusion marker mixed venous oxygen saturation (SvO_2_), measured using a blood gas analyzer.

### Hemodynamic monitoring

**Figure 1:**
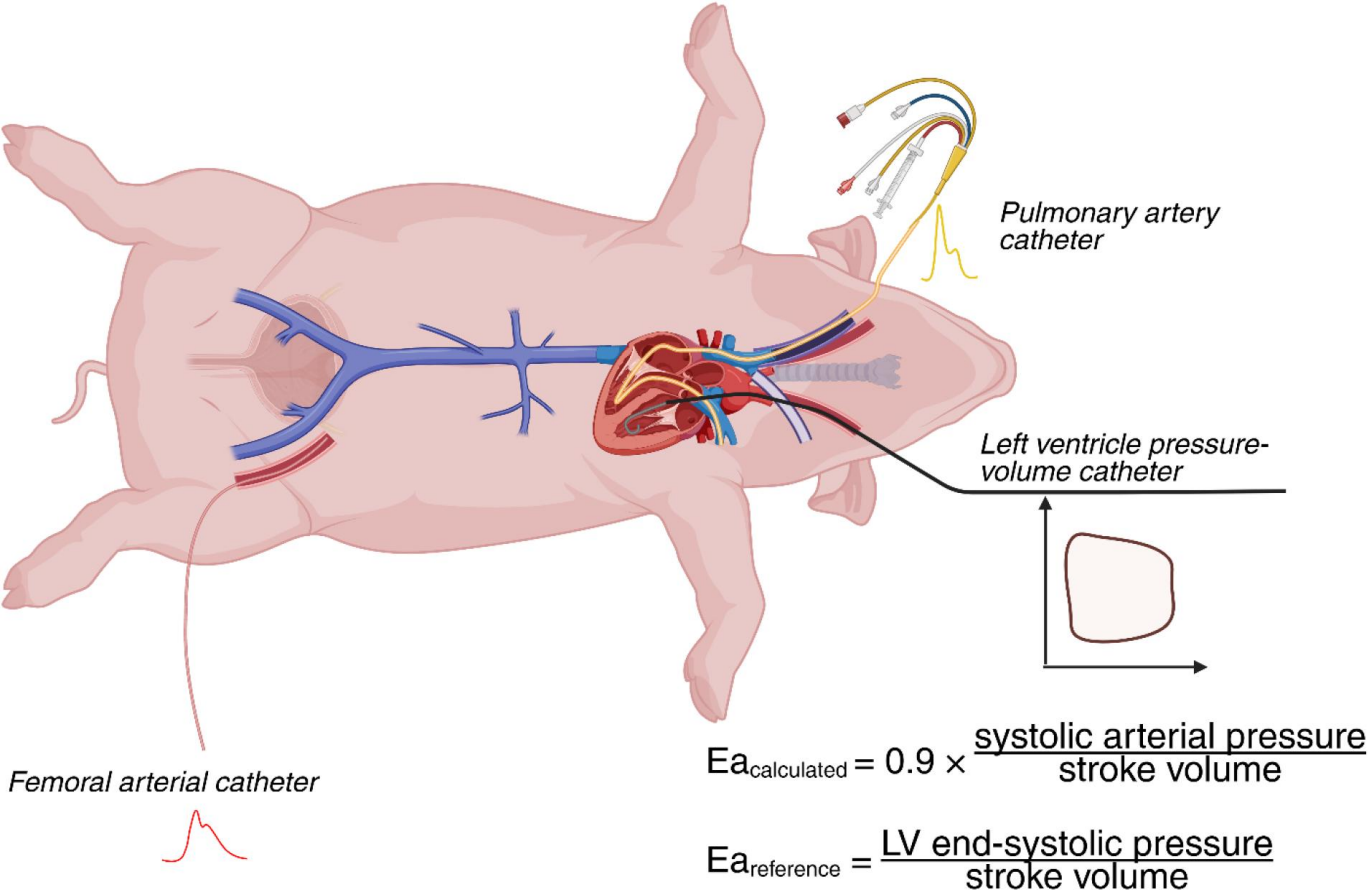
Animal instrumentation. Animals were instrumented prior to CS induction with a pulmonary artery catheter, a left ventricle pressure–volume admittance catheter, and a femoral arterial catheter. Arterial elastance (Ea) was calculated as described.

Briefly, cardiac output was obtained by the standard bolus thermodilution technique using a pulmonary artery catheter with three 10 mL boluses of cold saline injection into the right atrium, and the thermistor at the distal tip of the catheter recorded the resulting temperature-time curve. The average of three consecutive measurements with less than 10% variation was used. These cutoffs were chosen as they result in stable LV CS with marked contractile dysfunction, elevated afterload, elevated filling pressures, hypotension, and hypoperfusion without the need for immediate treatment escalation (1). The animals were instrumented prior to the induction of CS (Figure 1). Femoral artery pressure was continuously monitored. SV was estimated as cardiac output divided by heart rate. SVR was calculated using the standard formula. Pulmonary artery wedge pressure was also measured.

### Pressure–volume measurements

An LV PV admittance catheter (Transsonic, USA) was inserted through a carotid artery and used to obtain the standard reference Ea, as described elsewhere (1, 10). Briefly, the admittance-based PV catheter recorded instantaneous LV pressure and volume at high temporal resolution throughout each cardiac cycle. Ea was computed automatically using the Labchart 8 Pro software (AdInstruments, Australia). The automated software works as follows. ESP was identified as the LV pressure at the point of minimal LV volume, corresponding to the upper-left corner of the PV loop. SV was derived as the difference between the end-diastolic and end-systolic volumes. Reference Ea was then computed as ESP/SV, representing the slope of the line connecting the end-diastolic PV point to the end-systolic PV point and reflecting the net arterial load imposed on the LV per unit of ejected volume. LV end-systolic elastance (Ees), reflecting LV chamber contractility, was calculated from the PV-loop data as LVESP/(ESV−V0), with V_0_ derived from baseline PV-loop calibration and kept constant across serial measurements. Ventricular–arterial coupling (VAC) was then calculated as Ea/Ees, thereby relating arterial load to LV contractile function. LV end-diastolic pressure was also measured.

**Figure 2:**
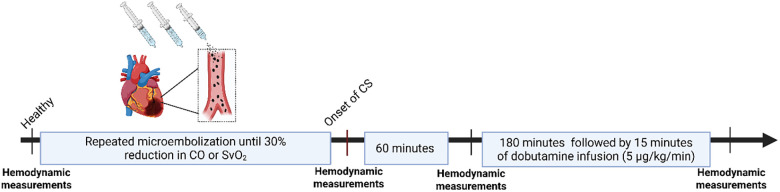
Study design. Hemodynamic measurements were first obtained at a healthy baseline. CS was then induced by repeated injections of microspheres into the left main coronary artery until either CO or SvO₂ had decreased by 30%. Measurements were repeated immediately after successful induction of CS, again after 60 min of untreated CS, and finally after 15 min of dobutamine infusion following an additional 180 min of untreated CS.

Ea_estimated_ was calculated using the formula 0.9 × systolic femoral pressure/SV (8). The agreement between estimated and reference Ea was evaluated at the following four timepoints: (i) in a healthy state, (ii) immediately following onset of ischemic CS, (iii) following 60 min of untreated ischemic CS, and (iv) after 15 min of an intravenous infusion of dobutamine 5 µg/kg/min after 3 h of untreated CS (Figure 2). Data analysis and graphical representations were performed using R software (version 4.2.1, RStudio, PBC) with Pearson correlation [95% confidence intervals (CIs)] and illustrated by Bland–Altman analysis stratified by predefined experimental timepoint separately at healthy baseline, CS onset, established untreated CS, and dobutamine-treated CS, reporting mean bias and 95% limits of agreement (LoA) after the exclusion of observations lying beyond 1.5 × IQR. Two-sided *P-*values <0.05 were deemed significant. Data are summarized as mean ± SD.

## Results

### Hemodynamic response to coronary embolization

Coronary embolization resulted in a marked hemodynamic deterioration consistent with ischemic CS (Table 1). Cardiac output fell from 4.2 ± 1.0 to 2.6 ± 0.4 L·min⁻^1^ (*P* < 0.001), driven primarily by a reduction in stroke volume from 71 ± 20 to 39 ± 9 mL (*P* < 0.001), with only a modest compensatory increase in heart rate. Ea increased while Ees decreased, resulting in increased VAC and indicating marked ventricular–arterial uncoupling during acute CS. Mean arterial pressure declined from 93 ± 13 to 71 ± 12 mmHg (*P* < 0.001), accompanied by a rise in LV filling pressures reflecting combined forward failure and backward congestion. Systemic vascular resistance increased markedly from 1,756 ± 534 to 2,928 ± 517 dyn·s·cm⁻⁵ (*P* < 0.05), indicative of compensatory sympathetic vasoconstriction. Mixed venous oxygen saturation fell from 58 ± 8% to 39 ± 7% (*P* < 0.001), confirming tissue hypoperfusion. Following 60 min of untreated CS, hemodynamic parameters remained largely unchanged from CS onset, with the exception of a partial reduction in SVR and increased heart rate. During dobutamine-treated CS, cardiac output increased and SVR decreased, while VAC improved but remained elevated compared with baseline.

**Table 1:** Hemodynamic values.

*N* = 20	Healthy	Onset of CS	Established untreated CS	Dobutamine-treated CS
Cardiac output (L min^−1^)	4.2 ± 1.0	2.6 ± 0.4^‡^	2.6 ± 0.6^‡^	5.1 ± 1.1
Stroke volume (mL)	71 ± 20	39 ± 9^‡^	38 ± 11^†^	54 ± 13*
Heart rate (bpm)	62 ± 18	69 ± 22*	75 ± 27^†^	99 ± 29^‡^
Mean arterial blood pressure (mm Hg)	93 ± 13	71 ± 12^‡^	70 ± 12^‡^	77 ± 13^†^
Systolic arterial blood pressure (mm Hg)	125 ± 15	95 ± 14^‡^	95 ± 15^‡^	112 ± 24*
Diastolic arterial blood pressure (mmHg)	75 ± 14	58 ± 13^‡^	58 ± 12^‡^	58 ± 9^†^
Pulmonary artery wedge pressure (mmHg)	7 ± 2	12 ± 3^‡^	13 ± 4^‡^	13 ± 3^‡^
Systemic vascular resistance (dyn s^−1^ cm^5^)	1,756 ± 534	2,928 ± 517*	2,060 ± 677*	1,175 ± 329*
Mixed venous oxygen saturation (%)	58 ± 8	39 ± 7^‡^	34 ± 6^‡^	56 ± 10
LV end-diastolic pressure (mmHg)	13 ± 5	18 ± 6^‡^	19 ± 6^‡^	20 ± 8^‡^
Ees (mmHg mL^−1^)	2.12 ± 0.56	1.11 ± 0.43^‡^	1.52 ± 0.89*	1.45 ± 0.54^†^
VAC	0.82 ± 0.27	2.39 ± 1.23^‡^	2.05 ± 1.24^‡^	1.32 ± 0.6^†^
Ea_estimated_ (mmHg mL^−1^)	1.72 ± 0.60	2.25 ± 0.61^‡^	2.25 ± 0.62^†^	1.95 ± 0.54
Ea_reference_ (mmHg mL^−1^)	1.73 ± 0.76	2.24 ± 0.89^†^	2.36 ± 1.02^†^	1.74 ± 0.69

Paired *t*-tests comparing hemodynamic values in the healthy state with the onset of CS, following 60 minutes of untreated CS (i.e. “established untreated CS”) and following dobutamine treatment respectively. ^‡^*P* < 0.001, ^†^*P* < 0.01, *P < 0.05.

### Overall

**Figure 3:**
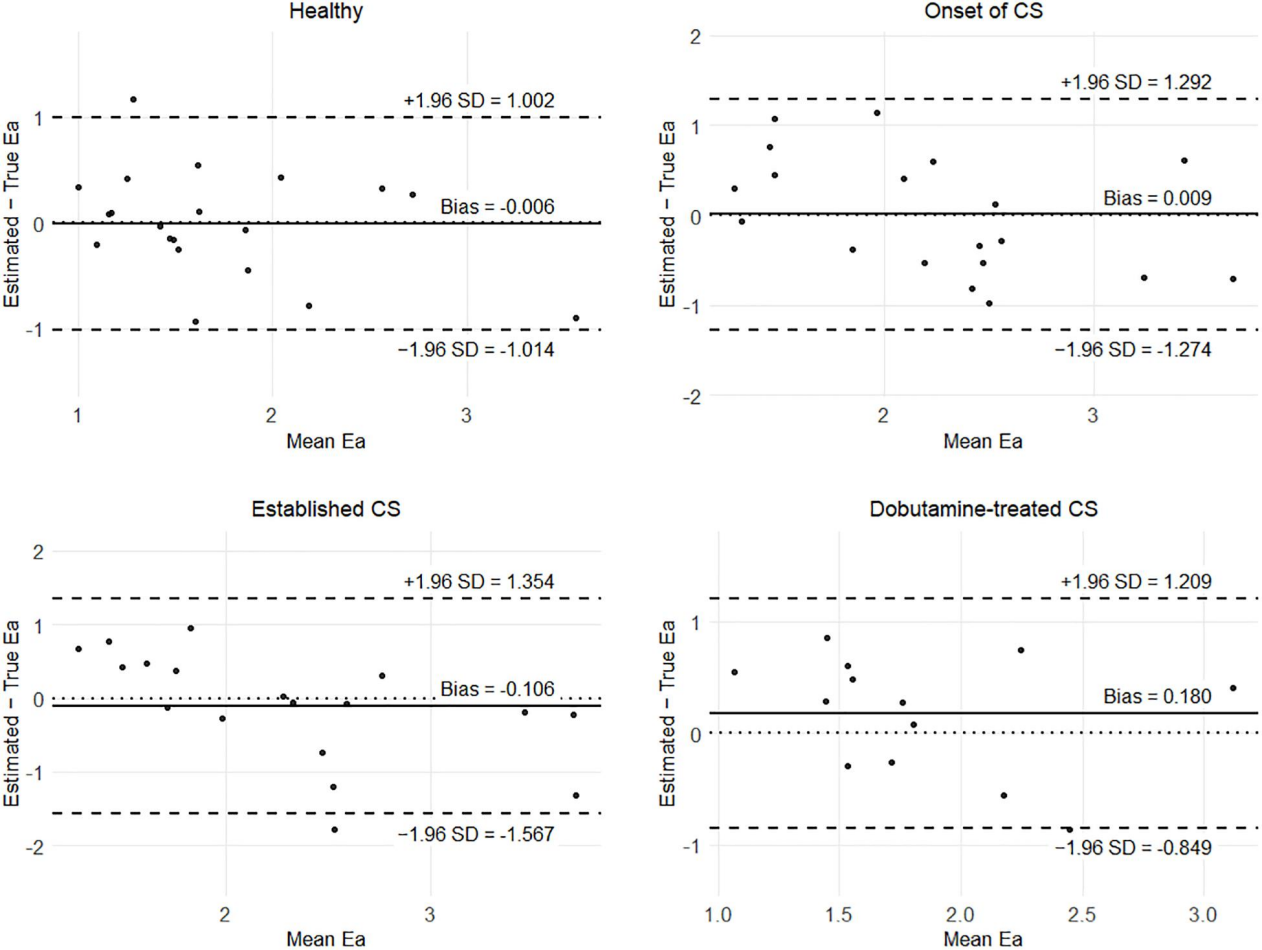
Time-specific Bland–Altman analysis of femoral pressure-derived Ea vs. PV-derived reference Ea. The Bland–Altman plots compare femoral pressure-derived Ea_estimated_ with pressure–volume-derived Ea_reference_ at the healthy baseline, onset of cardiogenic shock (CS), established untreated CS, and dobutamine-treated CS. The *y*-axis shows the difference between methods (Ea_estimated_−Ea_reference_), and the *x*-axis shows the mean of the two methods. Solid horizontal lines indicate mean bias, dashed lines indicate the upper and lower 95% limits of agreement, and dotted lines indicate zero difference.

**Figure 4:**
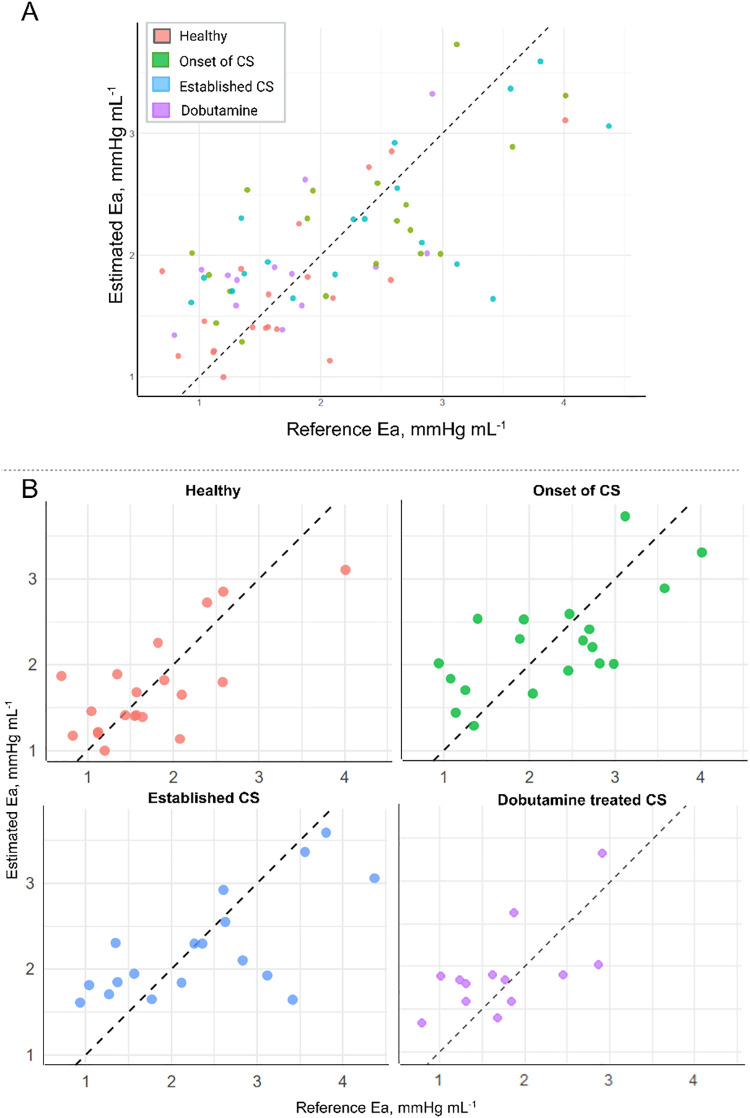
Linear regressions of estimated and reference Ea. **(A)** The linear regression analysis for the pooled observations for the estimated and reference Ea. **(B)** The linear regression of the estimated and reference Ea based on timepoints.

Both estimated and reference Ea increased from the healthy state to the CS states (Table 1). After pooling all 80 paired observations, the time-specific Bland–Altman analysis showed that agreement was preserved across the experimental states, but with state-dependent variation in bias and LoA (Figure 3). Bias was negligible at the healthy baseline and at CS onset, shifted toward slight underestimation during established untreated CS, and toward modest overestimation during dobutamine-treated CS. Estimated Ea reproduced the reference Ea with a negligible bias (2.04 ± 0.63 for Ea_estimated_ vs. 2.03 ± 0.88 mm Hg mL⁻^1^ for Ea_reference_) and a strong linear relationship (Pearson *r* = 0.72, 95% CI 0.58–0.82, *P* < 0.001) (Figures 4A,B).

### Healthy baseline

Before CS, Ea was nearly identical when calculated by the two methods (1.72 ± 0.60 vs. 1.73 ± 0.76 mm Hg mL⁻^1^), with an excellent correlation (*r* = 0.74, 95% CI 0.44–0.89, *P* < 0.001) and no systematic bias on Bland–Altman analysis (bias −0.006 mm Hg·mL⁻^1^; LoA −1.014 to 1.002), indicating that the estimate is accurate under normal ventricular–arterial coupling.

### Onset of ischemic cardiogenic shock

Ea increased immediately after coronary embolization. Ea was nearly identical when calculated by the two methods (2.25 ± 0.61 vs. 2.24 ± 0.89 mm Hg mL⁻^1^), with a still-robust correlation (*r* = 0.68, 95% CI 0.32–0.87, *P* = 0.001). The slight overestimation bias (+0.01 mm Hg mL⁻^1^) remained within the predefined LoA (+0.009 mm Hg·mL⁻^1^; limits of agreement −1.274 to 1.292).

### Established untreated shock

At the end of the 60-min period of untreated CS, the model retained good fidelity (2.25 ± 0.62 vs. 2.36 ± 1.02 mm Hg mL⁻^1^); correlation strength was maintained (*r* = 0.69, 95% CI 0.32–0.87, *P* = 0.002) with a modest underestimation bias (−0.106 mm Hg·mL⁻^1^). The LoA were widest at this timepoint (−1.567 to 1.354 mm Hg·mL⁻^1^), indicating greater individual-level variability during established untreated CS.

### Dobutamine-treated cardiogenic shock

Following inotropic support with dobutamine, both Ea_estimated_ and Ea_reference_ declined. The correlation between the methods remained statistically significant (*r* = 0.63, 95% CI 0.12–0.88, *P* = 0.020), though slightly attenuated compared to earlier timepoints with modest overestimation (bias +0.180 mm Hg·mL⁻^1^; limits of agreement −0.849 to 1.209).

## Discussion

Elevated afterload is a well-established aggravating factor in ischemic cardiogenic shock, as it increases myocardial oxygen demand and further compromises forward flow in the failing ventricle (2, 3). Vasoactive agents commonly used in shock management may either exacerbate or alleviate this burden depending on their pharmacological profile. Thus, the ability to monitor afterload continuously and with minimal invasiveness may provide important prognostic and therapeutic guidance. Our findings support that femoral pressure-derived Ea may serve as a reliable surrogate for invasively measured Ea in this context, potentially enabling clinicians to detect detrimental increases in afterload in real time and tailor therapy accordingly. Additional markers, such as Ees and VAC, further clarify the hemodynamic phenotype of ischemic CS. While Ea reflects the arterial load imposed on the ventricle, Ees reflects LV chamber contractility; VAC integrates these two components. Thus, an increase in Ea may be tolerated if matched by preserved or increased Ees, whereas a simultaneous increase in Ea and reduction in Ees produces ventricular–arterial uncoupling. In the present study, acute CS was characterized not only by increased Ea but also by a marked reduction in Ees and a pronounced increase in VAC. This supports the physiological interpretation that the failing LV became mismatched with the arterial system. Dobutamine increased cardiac output and reduced SVR, but VAC remained higher than in the healthy state, suggesting only partial restoration of ventricular–arterial coupling despite improved forward flow.

Several approaches have been proposed for estimating Ea and VAC at the bedside. Invasive PV-loop analysis remains the reference method because it directly relates LV end-systolic pressure to SV and allows simultaneous assessment of Ees. However, this approach is impractical in routine clinical shock management. Non-invasive and minimally invasive alternatives include echocardiographic estimation of SV combined with an arterial pressure-derived estimate of LVESP (12), MAP/SV-based approximations (13), arterial tonometry (14), and single-beat echocardiographic methods for Ees (15). These approaches increase clinical feasibility but introduce assumptions related to pressure site, LVESP approximation, volume measurement, and loading conditions. The results of this study demonstrate that the femoral pressure estimate of Ea remains reliable under healthy conditions, through the full dynamic evolution of acute CS, and during vasoactive treatment, when compared with direct Ea measurement using PV loops. These findings extend the previous validation of Ea estimation in hemodynamically stable patients (8) to the more extreme setting of an acute failing LV, confirming that the simple 0.9 × systolic femoral pressure/SV formula remains applicable even when SV decreases and SVR increases in concert. The use of femoral arterial pressure is also physiologically and clinically relevant. Radial arterial pressure may differ substantially from more central arterial pressure during shock and vasopressor therapy, and radial measurements may underestimate central pressure in critically ill patients (16, 17).

Because the estimated Ea correlated with the reference Ea with only small state-dependent shifts in bias, clinicians could use a single femoral artery tracing and thermodilution-derived SV to follow afterload continuously during the critical phase of developing CS, without the need for left heart catheterization. Importantly, the transition from negligible bias at baseline and CS onset to slight underestimation during established untreated CS and modest overestimation during dobutamine treatment suggests that acute changes in arterial compliance or wave reflection may alter the proportionality constant.

Notably, the correlation between the estimated Ea and the reference Ea remained significant even during inotropic support. However, the slightly elevated overestimation bias suggested that, while the model retained utility under pharmacological intervention, Ea estimation using femoral artery pressure recordings may be partially influenced by treatment-induced hemodynamic changes. The time-specific Bland–Altman plots also indicate that the agreement was the least precise during established untreated CS, where the LoA were widest despite preserved correlation. This suggests that femoral pressure-derived Ea may be reliable for tracking group-level and directional changes in afterload, while caution is warranted when interpreting single absolute values in prolonged untreated shock. A contribution from time-dependent PV catheter signal drift or calibration instability cannot be excluded, as PV catheter measurements are sensitive to signal drift and volume calibration procedures (18–20). However, such drift would not fully explain the shift toward overestimation during dobutamine treatment, suggesting that pharmacologically induced changes in vascular tone, wave reflection, or femoral-to-central pressure amplification may also have contributed.

The hemodynamic profiles at CS onset and established untreated CS were notably similar, with near-identical Ea values at both timepoints, but differences in HR and SVR. This possibly reflects the integrative nature of Ea, as, although Ea is largely load-independent, the marked rise in SVR at CS onset and the compensatory tachycardia at established CS have opposing effects on Ea, yielding comparable aggregate values despite distinct underlying hemodynamic compositions.

### Limitations

This study has several limitations. First, the experiment was performed in a controlled porcine model of ischemic CS, and the findings may not fully translate to human CS, where age, comorbidities, chronic vascular disease, and pre-existing reductions in arterial compliance may influence arterial load and pressure transmission. Second, Ea_estimated_ was derived from femoral systolic pressure and thermodilution-derived SV, and therefore still requires invasive arterial and pulmonary artery catheterization. Thus, the present method is less invasive than LV PV catheterization but is not fully non-invasive. Third, femoral pressure remains a peripheral surrogate of LV end-systolic pressure, and changes in vascular tone, wave reflection, and arterial compliance during CS or vasoactive therapy may alter the relationship between femoral systolic pressure and true LV end-systolic pressure. Furthermore, dobutamine-treated CS was assessed at a single dose and timepoint, and the performance of femoral pressure-derived Ea during other vasoactive regimens, mechanical circulatory support, or more prolonged shock states remains unknown.

Future research should test whether a dynamic scaling factor could further narrow the limits of agreement and whether non-invasive SV and blood pressure measurements could substitute for values measured using invasive procedures. Nonetheless, by demonstrating robust agreement across disease states, our study supports incorporating bedside estimated Ea using arterial pressure measurement into real-time assessment and therapeutic algorithms for CS.

## Data Availability

The raw data supporting the conclusions of this article will be made available by the authors, without undue reservation.
